# Address by W. W. H. Thackston, M. D., D. D. S.

**Published:** 1875-03

**Authors:** 


AMERICAN JOURNAL

OF

DENTAL SCIENCE.

Vol. VIII. THIRD SERIES—MARCH, 1875. No. 11.

### ARTICLE I.

*Address by Dr. W. W. H. Thackston.*

Gentlemen of the Virginia Association of Dental Surgeons:—

Your unprecedented kindness and personal regard has devolved upon me the duty of again presiding over your deliberations, and the agreeable office of once more uttering our anniversary greetings and congratulations.

We welcome you to this hall, and to the exercises of this occasion with the confident assurance that you have improved the lessons of our last meeting; and that you have in the year just closed, made a near approach to that "*excellence*" which we then attempted to portray as the great end and object of every true professional man, and we shall confess to a sense of disappointment if the work of this present session fails to reveal an advance and improvement commensurate with our opportunities and privileges, and co-equal with the progress and development that distinguish the other divisions and departments of science.

It is useless for me to remind the Virginia Association of Dental Surgeons that the present is a period of light, and of rapid movement in all the vocations of life; a resistless energy pervades the public mind, which imparts vigor, and gives character to the varied pursuits of men; and this principle tolerates no apathy, no supineness, and no lagging or halting by the way. We feel impressed with the conviction that these truths have been recognized by this Association; and that the transactions of this annual meeting will abundantly demonstrate the fidelity with which we have cultivated the field allotted to, or chosen by us.

We anticipate with interest and pleasure the committee reports upon the many subjects embraced in, or germain to our specialty of science; and as these reports and the discussions will eliminate and develope nearly, or quite all that is new, and all that is valuable, we propose in this introductory address to say nothing of theoretical or practical dentistry, and nothing of the several departments of science essential to the correct apprehension and successful practice of our specialty. As I have intimated, these subjects will be reported upon by our committees, and discussed by the Association: they form the staple of our literature, and constitute the *curriculum* of our colleges.

In selecting a topic for your entertainment and profit, we have been led to the choice of a subject, which, though fraught with considerations of gravest import to all composing a body which seeks to deserve, and which desires and claims recognition as a "liberal profession," has been strangely neglected and overlooked by our writers and teachers, our professors and public speakers.

The obligations of dentists to society, to the profession, and to *each other*, is the theme upon which we desire at this time to submit a few observations.

In the commercial, mechanical, agricultural, and other ordinary pursuits of life, the principle of individual and personal profit and advantage is the controlling incentive and distinguishing characteristic. In the rush, and whirl, and struggle for existence, for competence, and for wealth, the hideous principle of human selfishness is epitomized in the trite and common maxim, "every man for himself, and *devil* take the hindmost." Men, in a sense, become Ishmaelites; if the hand of each is not against his neighbor, it is only because that neighbor is too feeble to oppose his progress and thwart his plans, or too poor to pay tribute to his avarice and covetousness.

No controlling law of sympathy, no fraternal bond, and no accepted system of ethics, ties together or regulates the conduct of the busy throngs who fill the marts of trade, the shops of the artizan, or the fields of the farmer. It is true, that "trades unions," "commercial exchanges," and "farmers granges," are now seeking some amelioration, and some remedy for the ills, the inconveniences and losses incident to a system wanting in sympathy, in harmony, and in cooperation; a system that antagonizes each man with his colaborer or fellow craftsman, and leaves him *untaught*, *undisciplined* and *unrestrained* by any moral code or conventional usage, to rise or fall, to reflect honor, or to bring shame upon the business of his life.

What measure of success these several organizations have accomplished, or anticipate, we are not advised; we trust that for their own benefit, and the interests of society, they may do a good work. But as a general proposition, the merchant *will* yet sell all he can, without special regard for the rights, the claims or the interests of his fellow merchant; he does not consider it indecorous or unbusiness-like to decoy or draw away his neighbor's customers, or make the impression that *his* wares are cheaper and better, and his terms of trade more liberal and profitable to the buyer; and his fellow merchant retorts by crying, "shoddy," "humbug," and "gammon." The artizan underbids or disparages his fellow-craftsman as a "botch" and a "bungler." The manufacturer has his secret "recipes" and "formulas," his exclusive and peculiar processes and machinery, and always produces a better and cheaper article than his competitor can supply ; and the farmer, most manly and generous of them all, *will* nevertheless regard his own pigs as less mangy than his fellow-farmers, *his* horses the best, *his* cattle the fattest, and *his* implements and modes of cultivating the soil altogether superior to anything his clod-knocking, skillet-headed neighbor can exhibit or essay ; and he will undersell or oversell, and seek and avail himself of any advantage that shrewdness and sharp practice may secure, uncontrolled and unrestrained by anything save his individual, and often crude apprehensions of propriety of justice and of right. Such in brief is the style and character of the ordinary business pursuits of life, as distinguished from the several professions which claim to be "learned and liberal," and for that reason assume a higher position, and claim a higher plane in social esteem.

Away back in the dim and misty past, that epoch that immediately succeeded the dark ages, and that inaugurated the revival of letters, there was a bond of sympathy, a tie of brotherhood, and an unwritten law of kindness between the astrologers and alchemists, which, if we may credit the mouldy chronicles, and musty records of early history, was of binding force and universal application. As years and centuries passed, as letters were cultivated, as science began to crystalize from the elements of knowledge, and as vocations and callings began to separate themselves, and establish their metes and bounds, and prescribe their offices and functions, *rules* and *regulations* were adopted, having for their object the advancement and elevation of each department, and for the mutual benefit and advantage of the brotherhoods representing the divisions and specialties of science, denominated professions. As time wore on, as society became more cultivated and refined, as a higher civilization and a purer morality chastened the passions and elevated the characters of men, these rules and regulations assumed the form and exhibited the quality of a well digested and carefully arranged "code of ethics ;" the provisions and requirements of which have been acknowledged, and the practical observance and operation of which have developed, dignified and ennobled the several departments of science, and in large degree constituted the distinction between an acknowledged "liberal profession," and the several arts, trades and other pursuits of men.

As we propose to make a short and practical address, we shall for explanation and example refer to only one of the acknowledged "liberal professions," and for obvious reasons, we select the profession of medicine.

This grand old profession which embraces surgery, general and special; now hoary with years, and wisdom and virtue; ante-dating christianity, and recognized by the God man Himself as next in dignity and importance to His holy ministry, a profession cultivated, and taught, and practiced by a long line of the most illustrious men the world has seen; and now represented by the brightest intellects, the purest christians, and wisest philosophers of the nineteenth century. This profession has its "ethical code," which is taught, acknowledged, and obeyed by every medical man who respects himself and merits the recognition and respect of his fellow physicians and society. And why and wherefore? Because that code prescribes what the experience and wisdom of ages and the suggestions of benevolence, to say nothing of policy and expediency has demonstrated as best for the interests of science, best for the general good of society, and best for the individual success and usefulness of the professor and practitioner. Because it teaches the duty of physicians to their patients, their fraternal obligations to each other, and the fealty they owe to a humane and benevolent profession. Because it teaches and prescribes what is manly, and honorable, and gentlemanly; and reprobates what is mean, and low, and degrading in professional intercourse and practice, and because the spirit and letter of that "code" is to elevate the character, develop and improve the resources, and give to medicine a position and consideration second to no human calling in which men engage.

Who will say that the practitioners and professors of medicine and surgery do not stand the *peers* and recognized *equals* of the proudest and most exalted among men? And which among the learned professions lends more dignity, or confers more honor or consideration upon its members and followers? And why is it so? Not because of its antiquity, not wholly because of its enlarged benevolence and indispensable usefulness, not alone because of its great and varied learning; but because of *all* these combined with the distinguishing elevation of character, displayed by physicians in their professional intercourse with each other, and with society.

And now, gentlemen, to approach our objective point, how stands the case with *Dental Surgery*—a recognized, indispensable specialty of general medicine and surgery?

As a profession, we cannot boast the antiquity, or claim the learning that distinguishes medicine; it is only a few decades since dentistry moved out of the open domain of unlicensed art, and took its position in the line of accredited professions; some in this presence are contemporaries of its legal existence; for while dentistry was more or less successfully practiced centuries ago, it was not until the establishment of the first dental college, that the title and character of *profession* could be fairly or properly claimed.

Since the first regular and systematic course of college instruction was given, most marked and wonderful results have been realized in dental surgery; its rapid growth and development have at least kept pace with other sciences and professions; its advances, discoveries, and contributions to the sum of human knowledge, have at least demonstrated the "vigor of its youth, and the healthfulness of its organization." It has multiplied its resources, enlarged the scope of its usefulness, and greatly perfected its instruments and operations. It has in *anæsthesia*, given to humanity the great boon of the nineteenth century, and to science some of the most brilliant and startling discoveries in physiology and microscopical anatomy. It has created a special literature, and now sustains a number of well appointed and successfully conducted colleges.

From this standpoint, our progress would appear satisfactory, and our success most encouraging and gratifying; but, gentlemen, is it not true that we are lamentably deficient, and grievously derelict in understanding, appreciating, and observing the obligations accepted by all acknowledged professions? Especially, do we illustrate that fidelity to our profession, or cherish that respect and regard for the rights and the feelings of our fellow dentists, which is happily expressed in the maxim, "do unto others, as you would have them do unto you," and is it not true, that in our hurry and haste to make progress in the *material* departments of our calling, we have neglected and overlooked the moral attributes and considerations which are essential to a well rounded and symmetrical professional character?

I am happy in saying that dental surgery furnishes many examples of individual dignity and elevation of character; many conscientious and faithful observers of all the duties and obligations provided by the most rigid and exacting of ethical codes. Members we have, who instinctively, and from an innate sense of personal honor, measure up in all their acts and transactions to that standard of moral and professional excellence that seeks no improper or selfish advantage, nor stops short of the fulfillment of every moral and professional obligation. But unfortunately we are forced to acknowledge that such examples are not as general or universal as we could desire. We are a young and comparatively an undisciplined profession; a great deal of the leaven of quackery and charlatanism is yet to be neutralized or purged away; a great many of the "*tricks of trade*" still crop out and blur and blemish our professiona escutcheon.

By the "code of ethics" adopted by the Southern Dental Association, we are very fairly and thoroughly instructed as to our duties, deportment and obligations; and we most earnestly commend this "code" to your careful study and consideration, and would unhesitatingly recommend its adoption by this Association, with such additional provisions and requirements as experience and observation have demonstrated the necessity for.

The limits of such an address as we are now making, will permit no extended or elaborate discussion of the provisions and requirements of our professional "ethical code." Some however, of its most prominent and important features and conditions we desire to bring to your attention and impress upon your minds.

As the provisions of this code relate to society, we are required to be prompt in answering the calls, and meeting the demands of our patients; to be humane and tender in our practice and ministrations; discreet and gentlemanly in our bearing and deportment, giving to our patrons the benefit of our highest skill; but in no instance should we offer the inducement of a *warrant*, or the quack-like guarantee of infallible success. And now, gentlemen, just here allow me to ask, how common, and how general is this practice of warranting our operations? How often do these warrants and guarantees disappoint the expectations of our patients? How seriously do they compromise the character of our profession, and how often do they return to plague and damage their authors? The usage is discountenanced by the medical profession as wrong and impolitic in practice, and should be ignored and abandoned by all respectable dentists. If our attainments as scholars, and our skill as operators, if our reputations as gentlemen fail to inspire public confidence, rely upon it, that we are resorting to a most questionable and humiliating expedient, when we offer for patronage the inducement of a warrant.

But to resume, we are to respect the confidence reposed in us by our patients, and under no circumstances, expose the physical defects and misfortunes it is our office to repair and restore. It would be unprofessional and ruffianly, for a dentist to proclaim the edentulous mouth of his lady patient, or to exhibit as a trophy of his skill, the artificial substitutes he had been employed to construct; and so in like manner, it would be a violation of the spirit, as well as the letter of the "code," to expose any condition or circumstance that would pain the feelings, or shock the delicacy of our patients.

The wives, and daughters, and sisters of our patrons, are entrusted to our care and protection while the subjects of our operations and treatment; and that confidence should be acknowledged by the most exact and scrupulous regard, and the observance of all the proprieties of refined and cultivated society. In no pursuit or profession of life, is demanded a higher or more rigid illustration of all the qualities that distinguish a virtuous, refined and elevated gentleman, than in the relations and intercourse of the dental surgeon. Our personal habits, the appointments of our offices, and our conversation and general deportment should be such, that the most fastidious, the most timid and delicate of our patients may never hesitate, or fear to place themselves under our personal or professional care. While we should display the requisite firmness for the faithful performance of all our offices and operations, no roughness, no rudeness and barbarisms are permitted or tolerated by our code.

The dentist who enhances the unavoidable discomforts of his operations, or awakens the disgust of his patients by coarseness or vulgarity, not only irreparably damages himself, but inflicts contempt and reproach upon his profession.

For the specialty of medicine and surgery that we have chosen as the business of our lives, we should cherish admiration and affection; we should not regard it as simply a means of livelihood, a path to competence or fortune, but we should love it as one of the benign institutions of advanced civilization; we should love it for the benefits it dispenses to suffering humanity, and for the material and substantial rewards it bestows upon its faithful students and practitioners, and thus loving it, we should cheerfully and indefatigably labor for its advancement. Those who have gone before us, who have laid the foundations and built the superstructure upon which we now stand, have placed us under obligations we may never fully discharge. We enjoy the advantage of all the learning, the inventions, discoveries and improvements that have been wrought out and accumulated by our predecessors, and it is but a just and reasonable requirement that we should labor for the common good and general benefit of a profession to which we are so largely indebted.

We can therefore consistently have and hold no secret methods or forms of treatment, no instruments or operations, which can be withheld from the profession; we can appropriate to our personal and *exclusive* use and advantage, no invention, discovery or advance in science. It is true, that by a sort of common consent, or tacit acquiescence, the *patenting* of strictly mechanical implements and appliances, has been sanctioned, and such articles have been adopted and employed by the regular professions; but beyond this point, no individual appropriation of a professional improvement or discovery can be permitted. Humanity, to say nothing of the claims of science, has interests which cannot be overlooked or disregarded. Imagine for a moment, the effect and result of a patent upon, and an individual appropriation of *vaccination*, of *quinine*, of *chloroform*, of *morphia* and *mercury;* a monopoly of these and a thousand other indispensable remedies held by some merciless shylock, or soulless corporation? Think for a moment of the outrages and extortions, the frauds and corruptions of the "rubber patent;" frauds and exactions which have harried and worried the dentists of the country beyond endurance, and corruptions that have reached even the temple, and soiled the pure ermine of justice.

The protection of society, no less than the interests and reputation of science, demand that this *secret* and *patent system* be confined within the narrowest possible limits, in all recognized "liberal professions."

The professional man who makes a valuable discovery, or invents a useful instrument, and cheerfully dedicates it to public use and enjoyment, has his reward, and finds his recompense in the consciousness of a generous action, nobly performed, in the distinguishing esteem and consideration of his brotherhood, of society, and of posterity.

Who would to-day exchange the honor and immortality of Harvey, or of Jenner, and in our own profession, of Barnum, and a thousand other unselfish and elevated discoverers and inventors, for all the gold, and all the shame that has been gathered by Bacon and his company, from all their fraudulent patents and corrupt and collusive suits at law? So much, gentlemen, for secret remedies, secret methods of operating, and professional patents. We will now ask your consideration of another feature of the "code."

In communicating with the public, in announcing our purpose to practice, a change of office or residence, a modest card of a few lines, *is the correct thing.* The publication of long advertisements, the posting of great show bills, supplemented by a long list of certificates and testimonials from clergymen and other public and private citizens, setting forth wonderful and peculiar instruments and operations, extraordinary skill and great cheapness of terms, are practices utterly derogatory of professional character; and are reprobated and discountenanced by every principle of good taste, and every sentiment of personal dignity and professional honor. Such things are the devices of quacks and charlatans, of showmen and sharpers. No man of science so demeans himself while in the pale of professional recognition, or so demeaning himself, fails to forfeit the respect and esteem of his fellows, and the good opinion of refined and cultivated society. In our conduct and bearing before the public especially, we should exhibit respect and consideration for each other. We know the tendency and too common effect of sharp competition and ungenerous rivalry in practice; we know the temptation to disparage, or "damn with faint praise," the competitor who may appear to stand in our way, or obstruct our progress; but no sadder or more fatal mistake, no blunder more criminal can be committed, than for a professional man to habitually belittle, dispraise, or calumninate his fellow-dentist, or fellow-physician. Such conduct most usually defeats its object, and is sooner or later sure to bring upon its author the ret ribution of a lowered esteem, and impaired confidence in his own capacity and integrity.

We should be exceeding cautious how we criticise the modes of practice, and the operations of other dentists, particularly when we are consulted by those who have been their patients. We should discourage, and be slow to give countenance or credit to the complaints, the gossip, and censures so commonly indulged by parties who often from unworthy motives, seek a change of professional service, and desire to justify their conduct by defaming the dentist or physician who has faithfully, and to the best of their ability, answered all their calls and met their demands. We should not only feel it a duty, but find pleasure in *commending* the attainments, and *complimenting* the work of our fellow-dentists whenever we can conscientiously do so.

If our brother should fail, as all, even the most distinguished and skilfull *must* sometimes fail, we should explain and excuse his misfortune. Unfailing and unvarying success is the boon of no mortal man, and none, save the unprincipled charlatan, ever asserts such a pretension.

In our intercourse with each other, we should be kind, cordial and fraternal; we should ever hold ourselves ready to sustain a brother in the right, and to aid him by counsel, assistance and advice. We should open our offices, our laboratories and libraries, and freely and frankly interchange sentiments, opinions and convictions upon all professional subjects. The aged and experienced, should by kindly offices and prudent counsels, sustain and encourage the younger members of the profession; and the juniors should cherish and display a becoming deference and respect for those, whose long years of service and devotion have given to dental surgery its present consideration and importance. When necessary, or when requested to do so, we should cheerfully assist each other by consultation, or by taking part and assuming responsibility in any difficult or delicate operation. We should render each other, and the families of dentists while under the paternal roof, all necessary professional service, without the usual or specific fee. Where the use of any considerable amount of valuable material is demanded, no dentist who has the least respect for himself, will permit you to furnish it without compensation; and should your brother operator tender you an honorarium, or token of his appreciation of your skill and service, you may with consistency and propriety accept his offering; *but in no instance* are you to regard physicians who reciprocate professional services, or dentists and their families, the subjects of regular professional fees. And this, gentlemen, brings us to the last proposition we shall discuss in this address; to that prolific and unfailing source of discord and disagreement, of strife and estrangement in all professions, but perhaps more than any other, in our own: the subject of "fee tariffs."

We have sometimes thought it a great misfortune to humanity, and a great draw-back and impediment to the several professions, that there could be no *legalized minimum* scale of fees to which all practitioners should be rigidly held; that is, that it should be penal or punishable for any practitioner to underbid the prescribed rates of charge. Such a statute would certainly lop off, and prune away many excrescences, and much of the *fungi* that now blemish the character and impair the vitality of all professions. It would cure a great deal of pettifogging in law, a great deal of fanaticism, imposture and heresy in theology, and a great deal of quackery and humbug in medicine and surgery. But as we can have no statute law regulating professional fees, our next and only apparent remedy, is in conventional or professional law and usage. Now, gentlemen, we are no admirer or advocate of *mere fancy operations* in our specialty; we are no apologist of extravagant or exorbitant fees for our service; but as you, and the whole profession know, we have always urged the highest attainments, and the faithful exercise of the greatest skill for the benefit of our patients and patrons ; we have endeavored to teach and to stimulate your efforts by the conviction, that *excellence* and *superiority in practice* is the great demand of science, and of enlightened society.

While our profession is a charitable and beneficent one, we cannot as a general rule practice "*con amore*," or gratuitously ; we must have that profit and compensation that will meet and provide the wants and needs of life ; and it is unnecessary for me to tell *you*, that *low* and *cheap* fees, as popularly understood, are not compatible or consistent with good and faithful work. You know that a *low* "*fee tariff*" can only be maintained by hasty and imperfect work, and by the employment of most inferior implements and materials, and that the inevitable effect of such practice, is to arrest all progress, to degrade and debauch our profession, to impair our self-respect, and to diminish or forfeit the confidence and esteem of society.

We know of no one thing, we can imagine no single act of a professional man, that will so promptly and certainly bring upon him the distrust and contempt of society, as his cheapening the money value of his operations as a bid for patronage. The universal and very sensible conclusion is, "if the *pay is poor*, the *practice must be equally poor*."

A just regard for all the proprieties, equities and provisions of the ethical code, demand that an acknowledged and accredited dentist should adopt a "fee tariff" that will afford him fair and liberal compensation for the best and most faithful service he can render. When practicing in a town or city, or when elsewhere in competition with regular and respectable practitioners, he can with propriety do nothing less than accept the usual or established fees of the locality. But it is optional, and not in contravention of the spirit or letter of the code, for a physician or surgeon to adopt a *higher* "*fee tariff*" than had previously obtained in his field of practice. If his greater learning and superior skill can sustain such advance; should an appreciative community acknowledge his claims to enhanced compensation, he is *fairly* and *justly* entitled to the advantage. The demand and acceptance of higher fees can work no damage to his fellow-practitioner, or to the profession; but great good to both by the *stimulus* that generous compensation always gives to progress and improvement. But *cheapening*, undercharging, or more properly expressed, *underbidding*, to secure patronage and practice, is prohibited by every consideration of policy and propriety, and by every sentiment of professional honor and decency.

The comments and observations we have submitted upon the subject of fees and charges, have of course no application to what is known as eleemosynary, or charity practice; such demands all professions recognize, and accept for reward, the consciousness of a sacred duty cheerfully performed.

Such, gentlemen, is the spirit, and such some of the requirements and prominent features of that ethical code which will give dignity and character to your chosen pursuit, and which in all the professional relations of life, should distinguish you from the artizan and trader.

As you accept the provisions of this code, as you are imbued with its spirit, and illustrate its principles, so will you promote the best interests of society, and establish your individual claims to recognition as members and ornaments of a liberal and elevated profession.

